# Correlation between serum soluble ASGR1 concentration and low-density lipoprotein cholesterol levels: a cross-sectional study

**DOI:** 10.1186/s12944-023-01910-3

**Published:** 2023-09-04

**Authors:** Qin Luo, Jingfei Chen, Yingjie Su, Panyun Wu, Jiangang Wang, Zhenfei Fang, Fei Luo

**Affiliations:** 1 Department of Cardiovascular Medicine, The Second Xiangya Hospital, Central South University, Changsha, Hunan China; 2 Research Institute of Blood Lipid and Atherosclerosis, the Second Xiangya Hospital, Central South University, Changsha, China; 3 Reproductive Medicine Center, Department of Obstetrics and Gynecology, The Second Xiangya Hospital, Central South University, Changsha, Hunan China; 4 Department of Emergency Medicine, The Affiliated Changsha Central Hospital, Hengyang Medical School, University of South China, Changsha, China; 5 Department of Health Management, The Third Xiangya Hospital, Central South University, Changsha, 410013 Hunan China

**Keywords:** ASGR1, Cholesterol metabolism, Low-density lipoprotein cholesterol, Atherosclerotic cardiovascular disease, Lipid metabolism

## Abstract

**Background:**

Recent studies have shown that loss-of-function mutations in hepatic asialoglycoprotein receptor 1 (ASGR1) are associated with low levels of circulating cholesterol and a reduced risk of coronary artery disease (CAD). In contrast to ASGR1 on the hepatocyte membrane, serum soluble ASGR1 (sASGR1) is a secreted form that has been detected in circulation. However, the functions of serum sASGR1 are unclear. This study aims to investigate the relationship between human serum sASGR1 concentration and low-density lipoprotein cholesterol (LDL-C) levels.

**Methods:**

In a cohort of 134 participants who underwent coronary angiography examination, basic information was recorded, and blood samples were collected for biochemical testing. The serum sASGR1 concentration was determined by ELISA kits. The relationship between sASGR1 concentration and LDL-C levels was examined using linear regression models and interaction tests. Univariate and multivariate analyses were used to identify clinical variables that affect sASGR1 levels.

**Results:**

After adjusting for potential confounders such as age, sex, BMI, and statin use, the serum sASGR1 concentration was positively correlated with LDL-C levels (β = 0.093, 95% CI: 0.04 to 0.14, *P* < 0.001). Subgroup analysis and interaction tests showed that the effect of serum sASGR1 concentration on LDL-C levels was significantly influenced by hypertension status (*P* for interaction = 0.0067). The results of a multivariate linear regression analysis incorporating age, serum TG, LDL-C, nonesterified fatty acid (NEFA), white blood cell counts (WBCC), and fibrinogen revealed that LDL-C (β = 1.005, 95% CI: 0.35 to 1.66, *P* = 0.003) and WBCC (β = 0.787, 95% CI: 0.41 to 1.16, *P* < 0.0001) were independent influencing factors for serum sASGR1 levels.

**Conclusions:**

The serum sASGR1 concentration was positively correlated with LDL-C levels. In addition, hypertension status significantly affected the effect of serum sASGR1 on LDL-C levels. This study provides some research ideas for clinical doctors and researchers, as well as some references for additional research on serum sASGR1.

## Introduction

Atherosclerotic cardiovascular disease (ASCVD) is still one of the diseases with the highest mortality and disability rates in the world. Many studies have shown that abnormal lipid metabolism, especially high levels of low-density lipoprotein cholesterol (LDL-C), is a causal risk factor for ASCVD [1, 2]. Cholesterol-lowering therapy can significantly reduce the risk of cardiovascular events [3–5]. A meta-analysis showed that for every 1 mmol/l reduction in LDL-C, the risk of major vascular events decreased by 21% [6]. However, even with the use of high-intensity statins for lipid-lowering, certain patients are still at risk for cardiovascular events, so called residual cardiovascular risk [7, 8]. Proprotein convertase subtilisin/kexin type 9 (PCSK9) inhibitors and statins, which are the most effective cholesterol-lowering medications, increase LDL receptor (LDLR) expression to lower LDL-C levels [9, 10]. However, they are ineffective in patients with homozygous familial hypercholesterolemia [11]. Therefore, it is of great significance to find new lipid-lowering targets and develop new drugs.

In 2016, a human genetics study by Nioi et al. found that loss-of-function mutations in hepatic asialoglycoprotein receptor 1 (ASGR1) were associated with low levels of circulating cholesterol and a reduced risk of coronary artery disease (CAD) [12]. Recently, Wang et al. found that inhibiting the expression of hepatic ASGR1 (hASGR1) or its binding to circulating asialoglycoproteins promotes cholesterol efflux into bile, resulting in a decrease of approximately 25% in circulating total cholesterol (TC) and LDL-C levels [13]. These studies suggest that hASGR1 is expected to be a potential new lipid-lowering target.

**Fig. 1:**
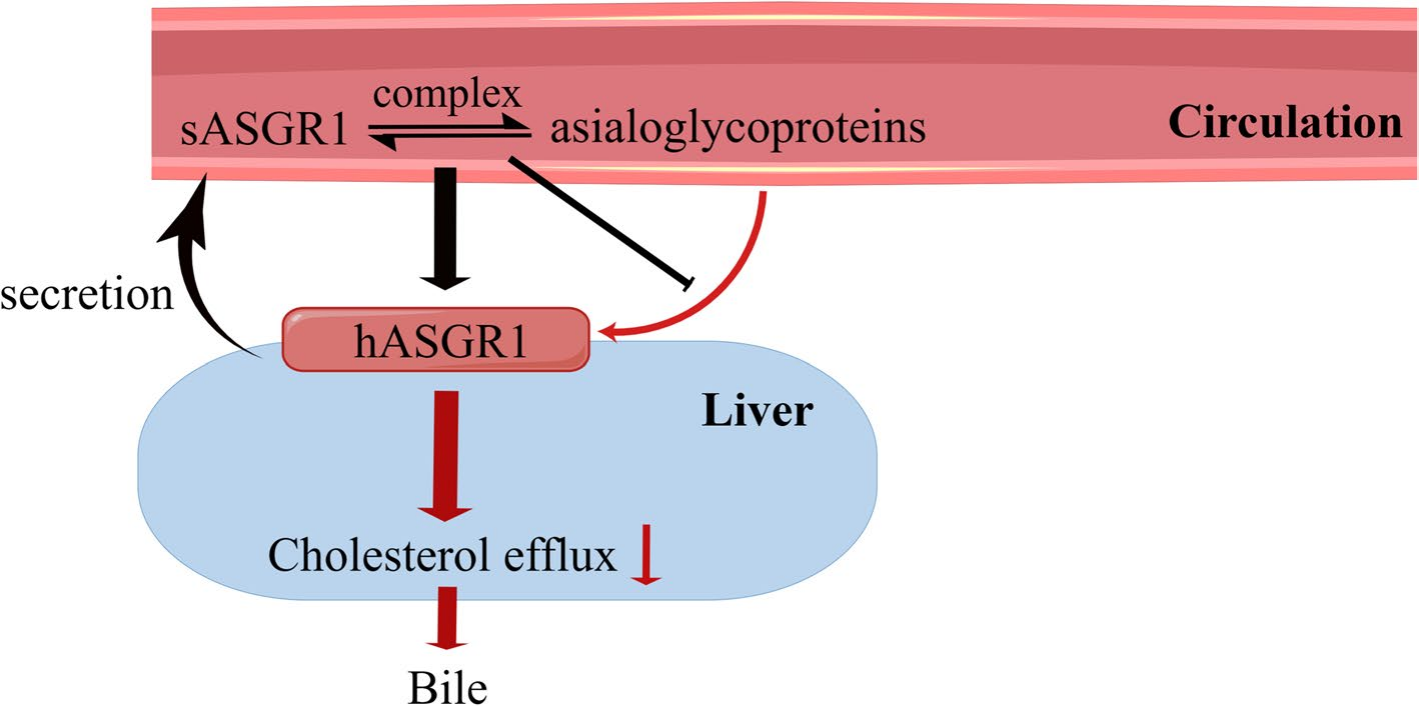
Relationship between serum sASGR1 and hASGR1. Circulating asialoglycoproteins bind hASGR1 on the surface of hepatocytes, ultimately resulting in reduced intracellular cholesterol efflux (red arrow). Circulating sASGR1 secreted by the liver competitively binds asialoglycoproteins with hASGR1. The sASGR1-asialoglycoprotein complex also enters the liver through hASGR1 on the surface of hepatocytes (black arrow)

ASGR1 was identified as two splice variants [14, 15]. In addition to the hASGR1 located on the hepatocyte membrane mentioned above, Liu et al. detected soluble ASGR1 (sASGR1) in human serum and HepG2 cell supernatant by western blot analysis, indicating that it was secreted by hepatocytes [15]. Structurally, compared to hASGR1, sASGR1 lacks 117 nucleotides, making it unable to encode a transmembrane domain [16, 17]. Interestingly, they further found that sASGR1 competitively binds circulating asialoglycoproteins with hASGR1, and the sASGR1-asialoglycoprotein complex is still captured by hASGR1 on the surface of hepatocytes and enters the cell [15], as shown in Fig. 1.

However, current research mainly focuses on the identity of ASGR1 as a liver surface receptor. The functions of sASGR1 are largely unknown. As an alternative splice form of ASGR1, sASGR1 may have a similar function to hASGR1 or be able to reflect the expression level of hASGR1. However, sASGR1 may also regulate lipid metabolism directly. The relationship between serum sASGR1 concentration and circulating cholesterol is unclear. Our study aims to investigate the relationship between human serum sASGR1 concentration and LDL-C levels.

## Materials and methods

### Study subjects

The study subjects were 134 patients who had undergone coronary angiography in the Department of Cardiovascular Medicine at the Second Xiangya Hospital of Central South University from September 2022 to April 2023. Patients with liver or kidney diseases, infections, autoimmune diseases, malignancies, and other severe diseases were excluded. The Second Xiangya Hospital’s ethics committee approved the study and determined that it complied with the ethical principles of the Declaration of Helsinki. Each patient gave informed consent.

### Diagnostic criteria

Clinical symptoms, ischemic electrocardiographic abnormalities, and coronary angiography demonstrating at least one main coronary artery stenosis of at least 50% were diagnostic criteria for CAD. The definition of acute myocardial infarction (AMI) is based on the fourth universal definition of myocardial infarction published by the ESC/ACC/AHA/WHF Task Force [18]. The diagnostic criteria for type 2 diabetes mellitus (T2DM) were fasting blood glucose ≥ 126 mg/dl (7.0 mmol/l) or 2-h postprandial blood glucose ≥ 200 mg/dl (11.1 mmol/l). Hypertension was diagnosed when resting systolic blood pressure was > 140 mmHg or diastolic blood pressure was > 90 mmHg on a different day.

### Clinical characteristics and biochemical measurements

The general clinical data of patients were obtained through the hospital’s electronic medical record system. Basic information such as the name, sex, age, BMI, and disease history of each patient was recorded. After fasting overnight, peripheral blood samples were collected. Serum was isolated from the blood samples and stored at -80 ℃ for subsequent analysis. Serum triglycerides (TG), TC, LDL-C, high-density lipoprotein cholesterol (HDL-C), lipoprotein (a) [Lp (a)], nonesterified fatty acid (NEFA), apolipoprotein-A1 (APO-A1), apolipoprotein-B (APO-B), alanine aminotransferase (ALT), aspartate aminotransferase (AST), albumin, creatinine, uric acid (UA), calcium, D-dimer, fibrinogen, high sensitivity troponin T (hsTNT), and N-terminal pro-brain natriuretic peptide (NT-proBNP) were determined using Hitachi 7600 series automatic analyzers in the Department of Laboratory Medicine of our hospital. Remanent cholesterol (RC) was estimated as TC minus LDL-C minus HDL-C. An automatic blood cell counter was used to measure white blood cell counts (WBCC). sASGR1 sandwich enzyme-linked immunosorbent assay (ELISA) kits (JL41965; Jianglai Biology, Shanghai) were used to measure the concentrations of serum sASGR1.

### Statistical analyses

The data were analyzed using EmpowerStats, statistical software (version 4.1, http://www.empowerstats.com, X&Y Solutions, Inc., Boston, MA), and R language (version 4.2.0, http://www.R-project.org, The R Foundation). In this study, the statistical threshold was set at *P* = 0.05.

Continuous variables are expressed as the mean ± standard deviation or as medians and quartiles based on the data distribution. Categorical variables are expressed as frequencies or percentages. We used univariate and multivariate linear regression models to evaluate the relationship between serum sASGR1 concentration and LDL-C levels as well as to identify clinical variables that affect sASGR1 levels. Both the crude and adjusted results from our models were provided in accordance with the STROBE statement’s standards [19]. Stratified linear regression models were utilized to conduct subgroup analyses, and likelihood ratio tests were utilized to identify changes and interactions between subgroups.

## Results

### Baseline characteristics of study participants

The baseline characteristics of the participants are shown in Table 1. There were 134 participants in total, with a mean age of 54.6 ± 13.8 years and a male participation rate of 47.76%. The number of patients with CAD was 84 (62.69%), and among them, 21 were diagnosed with AMI. Sixty patients (44.78%) used statins for lipid lowering. The proportion of smokers was 30.6%. The proportions of patients with T2DM and hypertension were 23.88% and 47.01%, respectively. The serum sASGR1 concentration ranged from 0.61 to 19.13 ng/ml (median: 2.49 ng/ml).

**Table 1:** Characteristics of study participants

	Patients (n = 134)
Age (years)	54.59 ± 13.78
BMI (kg/m^2^)	23.70 ± 3.29
TG (mmol/L)	1.46 (1.03, 2.06)
TC (mmol/L)	4.42 (3.88, 5.27)
LDL-C (mmol/L)	2.83 (2.36, 3.55)
HDL-C (mmol/L)	1.12 (0.96, 1.29)
RC (mmol/L)	0.43 (0.32, 0.56)
Lp (a) (mg/L)	174.70 (76.33, 382.10)
NEFA (mmol/L)	0.35 (0.25, 0.52)
APO-A1 (g/L)	1.02 ± 0.17
APO-B (g/L)	0.77 (0.65, 0.97)
WBCC (10^9^/L)	6.45 (5.34, 7.72)
ALT (μ/L)	17.20 (12.60, 25.38)
AST (μ/L)	17.70 (15.40, 25.10)
Albumin (g/L)	38.60 (37.05, 40.93)
Creatinine (μmol/L)	70.00 (59.00, 85.23)
UA (μmol/L)	316.00 (260.50, 382.50)
Calcium (mmol/L)	2.19 (2.12, 2.28)
Fibrinogen (g/L)	3.35 (2.90, 4.00)
D-dimer (μg/mL)	0.26 (0.20, 0.42)
Hs-TNT (pg/mL)	10.44 (5.33, 29.21)
NT-proBNP (pg/mL)	174.00 (52.45, 1008.00)
sASGR1 (ng/mL)	2.49 (1.71, 4.11)
Sex	N (%)
Male	64 (47.76%)
Female	70 (52.24%)
Statin use	
No	74 (55.22%)
Yes	60 (44.78%)
Smoke	
No	93 (69.40%)
Yes	41 (30.60%)
AMI	
No	113 (84.33%)
Yes	21 (15.67%)
CAD	
No	50 (37.31%)
Yes	84 (62.69%)
T2DM	
No	102 (76.12%)
Yes	32 (23.88%)
Hypertension	
No	71 (52.99%)
Yes	63 (47.01%)

The data displayed are the mean ± SD, median (interquartile range), or n (%)

*Abbreviations*: *BMI* body mass index, *TG* triglyceride, *TC* total cholesterol, *LDL-C* low-density lipoprotein cholesterol, *HDL-C* high-density lipoprotein cholesterol, *RC* remnant cholesterol, *Lp (a)* lipoprotein (a), *NEFA* nonesterified fatty acid, *APO-A1* apolipoprotein A1, *APO-B* apolipoprotein B, *WBCC* white blood cell counts, *sASGR1* soluble ASGR1, *ALT* alanine aminotransferase, *AST* aspartate aminotransferase, *UA* uric acid, *hs-TNT* high sensitivity troponin T, *NT-proBNP* N-terminal pro-brain natriuretic peptide, *AMI* acute myocardial infarction, *CAD* coronary artery disease, *T2DM* type 2 diabetes mellitus

### Univariate analysis

We utilized univariate linear regression analysis to assess whether the concentration of serum sASGR1 affects the level of LDL-C. As shown in Table 2, there was a significant difference in the effect of sASGR1 concentration on LDL-C (β = 0.10, *P* < 0.0001), which means that for every 1 ng/mL increase in sASGR1 concentration, the level of LDL-C rose by 0.1 mmol/L. In addition, TG, TC, RC, APO-B, NEFA, and the occurrence of CAD and AMI had statistically significant effects on LDL-C. Sex, age, BMI, statin use, smoking, HDL-C, Lp(a), APO-A1, and the occurrence of T2DM and hypertension were not associated with LDL-C.

**Table 2:** The results of univariate analysis

LDL-C	β (95% CI)	*P* value
Sex		
Male	ref	
Female	-0.14 (-0.46, 0.18)	0.3987
Age (years)	0.01 (-0.00, 0.02)	0.1842
BMI (kg/m^2^)	0.02 (-0.02, 0.07)	0.3270
Statin use		
No	0	
Yes	0.01 (-0.32, 0.33)	0.9622
Smoke		
No	0	
Yes	0.09 (-0.26, 0.44)	0.6116
AMI		
No	0	
Yes	0.53 (0.10, 0.97)	0.0184
TG (mmol/L)	0.21 (0.10, 0.33)	0.0003
TC (mmol/L)	0.78 (0.73, 0.83)	< 0.0001
HDL-C (mmol/L)	0.23 (-0.36, 0.81)	0.4511
RC (mmol/L)	0.76 (0.33, 1.19)	0.0007
Lp (a) (mg/L)	0.00 (-0.00, 0.00)	0.1280
NEFA (mmol/L)	0.95 (0.23, 1.67)	0.0113
APO-A1 (g/L)	0.70 (-0.41, 1.81)	0.2167
APO-B (g/L)	3.47 (3.25, 3.68)	< 0.0001
WBCC (10^9^/L)	0.10 (0.01, 0.19)	0.0319
CAD		
No	0	
Yes	0.36 (0.03, 0.69)	0.0340
T2DM		
No	0	
Yes	0.27 (-0.10, 0.65)	0.1574
Hypertension		
No	0	
Yes	0.17 (-0.16, 0.49)	0.3183
sASGR1 (ng/mL)	0.10 (0.06, 0.14)	< 0.0001

*Abbreviations*: *CI* Confidence interval

Other abbreviations are as shown in Table 1

### Relationship between sASGR1 and LDL-C

We further used multivariate analysis to evaluate the relationship between serum sASGR1 and LDL-C. Table 3 shows the crude model and three models after incorporating confounding factors. In the crude model, there was a significant difference in the impact of serum sASGR1 on LDL-C [β = 0.1, 95% confidence interval (CI): 0.06 to 0.14, *P* < 0.001]. Similarly, in the three models (model I, model II, and model III), after incorporating other covariates, such as age, sex, and BMI, serum sASGR1 was still the influencing factor of LDL-C.

**Table 3:** Multivariate analysis for the association between sASGR1 and LDL-C in different models

Outcome	Crude Model		Model I		Model II		Model III	
	β	*P*	β	*P*	β	*P*	β	*P*
	(95%CI)		(95%CI)		(95%CI)		(95%CI)	
sASGR1 (ng/mL)	0.100	< 0.001	0.098	< 0.001	0.096	< 0.001	0.093	< 0.001
	(0.06, 0.14)		(0.06, 0.14)		(0.06, 0.14)		(0.04, 0.14)	

Model I adjusted for age and sex

Model II adjusted for model I plus BMI, statin use, smoking status, and hypertension

Model III adjusted for models I and II plus AMI, NEFA, WBCC, CAD, and T2DM

### Subgroup analysis and interaction tests

As shown in Table 4, the interaction test showed that the effect of sASGR1 on LDL-C was significantly affected by hypertension status (*P* for interaction = 0.0067), while age, sex, BMI, and the status of CAD and T2DM were not statistically significant in the interaction test. Our results showed that the effect size of sASGR1 on LDL-C showed differences between hypertensive and nonhypertensive patients. In hypertensive patients, serum sASGR1 was positively correlated with LDL-C levels [β = 0.15, 95% CI (0.08, 0.23), *P* < 0.001], while this relationship could not be observed in nonhypertensive patients. In addition, we noticed that the effect of sASGR1 on LDL-C showed differences in CAD status and age, although the interaction test was not statistically significant. For non-CAD patients and patients younger than 60 years, serum sASGR1 was not significantly associated with LDL-C.

**Table 4:** Subgroup analysis for the association between sASGR1 and LDL-C

Variables	Effect size (95%CI)	*P* value	*P* for interaction
Age (years)			0.39
Below 60	0.06 (-0.01, 0.13)	0.09	
Over 60	0.09 (0.02,0.17)	0.02	
Sex			0.73
Male	0.08 (0.01, 0.14)	0.02	
Female	0.11 (0.02, 0.21)	0.02	
BMI (kg/m^2^)			0.39
< 24	0.07 (0.01, 0.13)	0.02	
> = 24	0.11 (0.01, 0.21)	0.04	
CAD			0.14
No	-0.03 (-0.17, 0.11)	0.66	
Yes	0.10 (0.04, 0.15)	< 0.001	
Hypertension			0.0067
No	0.04 (-0.04, 0.11)	0.33	
Yes	0.15 (0.08, 0.23)	< 0.001	
T2DM			0.53
No	0.10 (0.03, 0.16)	0.004	
Yes	0.13 (0.06, 0.19)	0.002	

The above model adjusted for age, sex, BMI, statin use, smoking status, hypertension, AMI, NEFA, WBCC, CAD, and T2DM

In each case, the model is not adjusted for the stratification variable

### The relationship between clinical variables and sASGR1 concentration

To identify other clinical variables that affect the concentration of sASGR1, we conducted a univariate linear regression analysis. As shown in Table 5, the concentration of serum sASGR1 is affected by age, serum TG, TC, LDL-C, NEFA, APO-A1, APO-B, fibrinogen, WBCC, and AST. Sex, BMI, statin use, smoking, serum HDL-C, Lp(a), ALT, albumin, creatinine, UA, calcium, D-dimer, hs-TNT, NT-proBNP, and the presence of T2DM, AMI, and hypertension were not found to be associated with serum sASGR1 concentration. We then performed a multivariate linear regression analysis (Table 6). Based on sample size, collinearity between independent variables, and univariate analysis results, we set age, LDL-C, TG, NEFA, WBCC, and fibrinogen as independent variables. The results showed that the effect of LDL-C level on the concentration of sASGR1 was statistically significant [β = 1.005, 95% CI (0.35, 1.66), *P* = 0.003]. Notably, WBCC was also an independent factor affecting sASGR1 concentrations [β = 0.787, 95% CI (0.41, 1.16), *P* < 0.0001].

**Table 5:** Univariable correlations of the clinical variables with the serum sASGR1 levels

Variables	β (95%CI)	*P* value
Sex		
Male	ref	
Female	-0.31 (-1.63, 1.02)	0.6511
Age (years)	0.05 (0.00, 0.10)	0.0466
BMI (kg/m^2^)	0.07 (-0.13, 0.27)	0.4985
Statin use		
No	0	
Yes	0.42 (-0.90, 1.75)	0.5337
Smoke		
No	0	
Yes	0.40 (-1.03, 1.83)	0.5827
AMI		
No	0	
Yes	1.84 (-0.36, 4.05)	0.1051
T2DM		
No	0	
Yes	1.52 (-0.01, 3.04)	0.0535
Hypertension		
No	0	
Yes	0.76 (-0.56, 2.07)	0.2629
TG (mmol/L)	0.65 (0.17, 1.12)	0.0082
TC (mmol/L)	1.55 (1.04, 2.07)	< 0.0001
LDL-C (mmol/L)	1.66 (1.03, 2.30)	< 0.0001
HDL-C (mmol/L)	1.28 (-1.10, 3.66)	0.2928
Lp(a) (mg/L)	-0.00 (-0.00, 0.00)	0.8501
NEFA (mmol/L)	6.26 (3.43, 9.08)	< 0.0001
APO-A1 (g/L)	6.73 (2.31, 11.15)	0.0035
APO-B (g/L)	7.01 (4.55, 9.48)	< 0.0001
Fibrinogen (g/L)	0.96 (0.38, 1.55)	0.0016
WBCC (10^9^/L)	0.86 (0.51, 1.20)	< 0.0001
ALT (μ/L)	0.03 (-0.01, 0.07)	0.1048
AST (μ/L)	0.03 (0.00, 0.05)	0.0242
Albumin (g/L)	0.16 (-0.03, 0.34)	0.0951
Creatinine (μmol/L)	0.01 (-0.03, 0.04)	0.7542
UA (μmol/L)	0.00 (-0.00, 0.01)	0.4062
Calcium (mmol/L)	0.97 (-3.13, 5.06)	0.6447
D-dimer (μg/mL)	-0.10 (-0.62, 0.41)	0.6959
Hs-TNT (pg/mL)	0.00 (-0.00, 0.00)	0.2316
NT-proBNP (pg/mL)	-0.00 (-0.00, 0.00)	0.8771

Abbreviations are as shown in Table 1

**Table 6:** Multiple regression analysis detecting independent contributors to sASGR1 levels

Variables	β (95%CI)	*P* value	Vif
Age (years)	0.009 (-0.03, 0.05)	0.676	1.185
TG (mmol/L)	0.274 (-0.16, 0.71)	0.211	1.149
LDL-C (mmol/L)	1.005 (0.35, 1.66)	0.003	1.242
NEFA (mmol/L)	2.405 (-0.20, 5.01)	0.07	1.229
WBCC (10^9^/L)	0.787 (0.41, 1.16)	< 0.0001	1.324
Fibrinogen (g/L)	0.005 (-0.66, 0.67)	0.989	1.465

Abbreviations are as shown in Table 1

## Discussion

In this study, we used a linear regression model to explore the relationship between serum sASGR1 and LDL-C levels. Univariate analysis showed a positive relationship between serum sASGR1 concentration and LDL-C levels (β = 0.1, *P* < 0.001). Similar results were still obtained in the multivariable linear regression models incorporating factors, such as age, sex, BMI, and smoking status.

Previous animal studies have shown that the binding of hASGR1 to asialoglycoproteins in the circulation determines the flow of hepatic cholesterol efflux into bile [13]. Inhibiting hASGR1 or its binding to circulating asialoglycoproteins will lead to increased cholesterol efflux, thereby reducing circulating TC and LDL-C levels [13]. Our study demonstrated a positive correlation between serum sASGR1 and LDL-C, which is similar to the relationship between hASGR1 and LDL-C, suggesting that the concentration of sASGR1 may reflect the protein expression level of hASGR1 to some extent. However, whether sASGR1 is directly involved in the metabolism of LDL-C remains unclear.

Subgroup analysis and interaction tests are helpful to evaluate the heterogeneity of populations with different characteristics. Our results showed that only hypertension status affected the effect of serum sASGR1 on LDL-C after grouping for age, sex, BMI, CAD, hypertension, and T2DM status. Through a PubMed literature search, only one study on ASGR1 and hypertension was obtained. This study showed that the single nucleotide polymorphism rs34870220 of ASGR1 was associated with the occurrence of essential hypertension [20]. The authors speculated that ASGR1 may be involved in vascular endothelial injury [20]. Because there are few similar reports at present, we cannot explain why the linear correlation between sASGR1 and LDL-C can only be observed in hypertensive patients. Additionally, we noticed that the effect of serum sASGR1 on LDL-C showed differences in CAD status, although the *P* for the interaction test did not reach statistical significance. The interaction *P* value is related to the overall sample size and the number of stratified individuals. Therefore, the insufficient number of non-CAD patients may be one of the reasons. In addition, the degree of dispersion of LDL-C levels in non-CAD patients was less than that in CAD patients (0.65 vs. 1.08 mmol/l), while serum sASGR1 may be affected by other unknown factors, showing a greater degree of dispersion in our results. This may also be one of the reasons for the lack of correlation between serum sASGR1 and LDL-C in non-CAD and nonhypertensive patients. However, we cannot rule out the possibility that there is no correlation between serum sASGR1 and LDL-C under healthy conditions.

Furthermore, we attempted to identify clinical variables that affect sASGR1 levels. Although univariate analysis suggests that a number of clinical variables are associated with sASGR1 levels, multivariate linear regression analysis shows that LDL-C and WBCC are independent influencing factors for sASGR1 levels. This is consistent with our results of setting LDL-C as the dependent variable, indicating a positive correlation between sASGR1 and LDL-C levels. WBCC is a widely recognized classic indicator of inflammation. This result seems to suggest that sASGR1 may be related to systemic inflammation. In fact, some basic studies have revealed a correlation between hASGR1, a membrane surface receptor, and inflammation [21–23]. However, the relationship between serum sASGR1 and inflammation is unknown, and further research is needed. In addition, previous studies have shown that the binding of circulating asialoglycoprotein to hepatic ASGR depends on calcium ions [24]. Our results indicate that the concentration of sASGR1 is not affected by serum calcium levels.

Since the relationship between hASGR1 and serum cholesterol was discovered in 2016, ASGR1 has attracted researchers’ attention mainly as a liver surface receptor. Although the presence of sASGR1 has been confirmed, there are few studies on serum sASGR1, especially investigating its relationship with blood lipids. This is the first investigation of the correlation between serum sASGR1 and LDL-C levels that we are aware of. In the follow-up study, the relationship between serum sASGR1 and CAD risk needs further investigation. The current study suggests that hASGR1 may become a future lipid-lowering target. Inhibiting hASGR1 or its binding to circulating asialoglycoproteins may reduce circulating cholesterol by promoting cholesterol efflux. Therefore, the application of ASGR1 inhibitors in populations with high expression of ASGR1 in the liver may be beneficial. However, there is still no simple and effective method to measure the expression level of hASGR1. Whether the concentration of serum sASGR1 reflects the expression level of hASGR1 deserves further study. In addition, the function of serum sASGR1 is unknown. Whether serum sASGR1 is also directly involved in the metabolism of LDL-C needs further study. Based on the research results of Liu et al. [15], it is intriguing to see whether the intervention of sASGR1 can directly achieve a lipid-lowering effect if entry of the sASGR1-asialoglycoprotein complex into the liver results in the same downstream effects as circulating asialoglycoprotein entry into the liver via hASGR1. Finally, our study provides some research ideas for clinical doctors and researchers, as well as some references for additional research on hepatic and serum ASGR1.

### Study strengths and limitations

Our study has some strengths. First, we investigated the variables affecting serum sASGR1 levels and the correlation between sASGR1 and LDL-C levels for the first time. Second, we used rigorous statistical adjustment models to minimize the unavoidable confounding factors of observational studies. Third, we observed a positive correlation between sASGR1 and LDL-C levels in CAD patients in the results of subgroup analysis rather than in non-CAD patients, which means that intervention with sASGR1 may have greater clinical significance.

There are some limitations to this study. First, this study is a cross-sectional survey; therefore, the correlation between research factors and outcomes is exploratory, and their causality needs to be further confirmed by prospective studies, although previous basic studies have revealed the causal relationship between hASGR1 and LDL-C. Second, the sample size of our single-center study is limited, so the results may not be generalizable.

## Conclusion

Overall, serum sASGR1 concentration was positively correlated with LDL-C levels. Hypertension status significantly affected the effect of sASGR1 on LDL-C levels. In addition, LDL-C levels and WBCC are independent influencing factors for serum sASGR1 concentration. Future research should focus on the function of serum sASGR1 and how it relates to CAD risk. It’s worth considering whether serum sASGR1 could become a potential lipid-lowering target like hASGR1.

## Data Availability

The datasets used and/or analyzed during the current study are available from the corresponding author upon reasonable request.

## Abbreviations

ASGR1: Asialoglycoprotein receptor 1
CAD: Coronary artery disease
hASGR1: Hepatic ASGR1
sASGR1: Soluble ASGR1
LDL-C: Low-density lipoprotein cholesterol
ASCVD: Atherosclerotic cardiovascular disease
PCSK9: Proprotein convertase subtilisin/kexin type 9
TC: Total cholesterol
AMI: Acute myocardial infarction
T2DM: Type 2 diabetes mellitus
TG: Triglycerides
HDL-C: High-density lipoprotein cholesterol
RC: Remanent cholesterol
Lp (a): Lipoprotein (a)
NEFA: Nonesterified fatty acid
APO-A1: Apolipoprotein-A1
APO-B: Apolipoprotein B
WBCC: White blood cell counts
ALT: Alanine aminotransferase
AST: Aspartate aminotransferase
UA: Uric acid
Hs-TNT: High sensitivity troponin T
NT-proBNP: N-terminal pro-brain natriuretic peptide
ELISA: Enzyme-linked immunosorbent assay
